# L-Cysteine Modified Chitosan Nanoparticles and Carbon-Based Nanostructures for the Intranasal Delivery of Galantamine

**DOI:** 10.3390/polym14194004

**Published:** 2022-09-24

**Authors:** Stavroula G. Nanaki, Konstantinos Spyrou, Pelagia Veneti, Niki Karouta, Dimitrios Gournis, Turki N. Baroud, Panagiotis Barmpalexis, Dimitrios N. Bikiaris

**Affiliations:** 1 Laboratory of Polymer Chemistry and Technology, Department of Chemistry, Aristotle University of Thessaloniki, 54124 Thessaloniki, Greece; 2 Department of Materials Science and Engineering, University of Ioannina, 45110 Ioannina, Greece; 3 Materials Science and Engineering, King Fahd University of Petroleum & Minerals, Dhahran 31261, Saudi Arabia; 4 Department of Pharmaceutical Technology, School of Pharmacy, Aristotle University of Thessaloniki, 54124 Thessaloniki, Greece

**Keywords:** modified chitosan, L-cysteine, galantamine, nanoparticles, drug delivery, carbon dots, porous carbon nanostructures

## Abstract

The present study evaluates the use of thiolized chitosan conjugates (CS) in combination with two fundamental carbon nanoforms (carbon dots (CDs) and Hierarchical Porous Carbons (HPC)) for the preparation of intranasally (IN) administrated galantamine (GAL) nanoparticles (NPs). Initially, the modification of CS with L-cysteine (Cys) was performed, and the successful formation of a Cys-CS conjugates was verified via ^1^H-NMR, FTIR, and pXRD. The new Cys-CS conjugate showed a significant solubility enhancement in neutral and alkaline pH, improving CS’s utility as a matrix-carrier for IN drug administration. In a further step, drug-loaded NPs were prepared via solid-oil–water double emulsification, and thoroughly analyzed by SEM, DLS, FTIR and pXRD. The results showed the formation of spherical NPs with a smooth surface, while the drug was amorphously dispersed within most of the prepared NPs, with the exemption of those systems contianing the CDs. Finally, in vitro dissolution release studies revealed that the prepared NPs could prolong GAL’s release for up to 12 days. In sum, regarding the most promising system, the results of the present study clearly suggest that the preparation of NPs using both Cys-CS and CDs results in a more thermodynamically stable drug dispersion, while a zero-order release profile was achieved, which is essential to attain a stable in vivo pharmacokinetic behavior.

## 1. Introduction

Galantamine (GAL) is a selective acetylcholinesterase (AChE) inhibitor that enhances cholinergic neurotransmission by inhibiting acetylcholine metabolism in the postsynaptic clefts [1,2]. It is used to treat the symptoms of Alzheimer’s disease (AD) by increasing the amount of a certain natural substance in the brain, which is needed for memory and thought [3]. In terms of medication regimens, even though different administration routes have been explored in the lab, only *per os* administration of GAL has been approved for clinical use [4]. After its oral intake, the drug shows high bioavailability with a fast onset (t_max_ 0.5–2h), while food intake does not compromise its efficacy [5,6]. Despite the fact that GAL is considered to be a well-tolerated and safe drug, the most frequent adverse effects, leading to treatment withdrawal, primarily comprise gastrointestinal symptoms associated with its oral administration, such as nausea, vomiting and diarrhea [3]. In addition, difficulties are associated with the characteristics of AD and patience treatment compliance, as well as the fact that GAL is not able to cross the blood–brain-barrier (BBB) efficiently when administrated peripherically (i.e., orally or parenterally), making its oral used unsuitable.

In this framework, the development of new, innovative drug delivery formulations for GAL, utilizing other means of administration, is significant. One alternative and highly promising route, which is not peripheral and is extremely suitable for AD patient treatment with AChE inhibitors (such as GAL), is through the intranasal (IN) cavity [7]. In general, IN administration appears to be a comfortable and simple method to bypass the BBB and deliver the medication straight into the brain [8]. Among its several advantages, IN administration provides: (1) a faster onset of action that can avoid the first-pass metabolism, (2) a reduction in several side effects associated with systematic administration, (3) accurate drug targeting, and (4) a convenient and patient-friendly route of drug administration [7,9,10,11]. However, despite its many benefits, there is still a lot of ground to cover when it comes to the implementation of IN and AD treatment. In this direction, several attempts are currently being made by various research groups.

The first study reporting GAL IN administration was published in 2012 by Li et al. [12]. In this work, the utilization of a novel flexible liposomal system resulted in significant improvements in GAL bioavailability (measured in terms of AUC) compared to the oral formulation. Additionally, the proposed system showed a good safety profile with no toxicity. However, even though the suggested liposomal system appeared to be promising, concerns regarding its stability, along with several other practical implementation issues (noted by the authors), diverted research attention to other solutions. As a result, two separate research groups, i.e., Hanafy et al. [13] and Bhattacharya et al. [14] explored alternative ways to address the same problem (i.e., successful GAL IN administration). In the former case, a nano-system comprising a cationic polymeric carrier, namely chitosan (CS), and GAL, was evaluated, while Bhattacharya and coworkers investigated the alleviation of AD symptoms in mice by intranasally administering a pro-drug form of GLN (i.e., memogain). In both cases, promising results were reported regarding GAL’s safety and efficacy. In a follow up study, conducted by Hanafy et al., the authors focused their attention on CS-based nanoparticles (NPs) [15]. The results showed that GLA-CS NPs can significantly lower the AChE levels in the brain, while boosting the cholinergic functioning. These findings clearly established CS as one of the most promising polymeric carriers for GAL’s IN administration [16].

In general, CS is a hemi-synthetic, cationic, linear, naturally occurring polysaccharide that is produced by the deacetylation of chitin and used in a variety of pharmaceutical formulation applications [17,18,19,20,21,22]. Its promising results regarding IN administration are due to its high stability, good biodegradability and biocompatibility, as well as its low toxicity [23,24]. Additionally, the mucoadhesive properties of CS enable its prolonged maintenance in the nasal cavity, thus leading to the expansion of the nasal–mucosal absorbance by simultaneously diminishing the mucociliary clearance [7,25]. However, despite its several advantages, the mucoadhensive properties of the pure (unmodified) CS make the polymer marginally suitable for IN administration [26]. The reason for this is the fact that nasal mucosal pH is approximately 5.5–6.5, and increases in rhinitis to 7.2–8.3, while a basic polymer such as CS is only mucoadhesive at limited acidic pHs. Additionally, as CS is only soluble at pH < 6, its usage in environments with a high pH (such as the nasal cavity) may lead to precipitation [27,28]. To overcome these drawbacks, several modifications to CS have been proposed, including trimethyl CS, carboxymethyl CS, thiolated CS etc. [29,30,31,32,33,34,35,36,37]. Among them, the thiolation of CS (i.e., its modification with agents with thiol groups, such as cysteine, thioglycolic acid, and 2-iminothiolane) has demonstrated several advantages compared to unmodified CS, including better mucoadhension and permeation, as well as enhanced gelling and inhibitory efflux properties [27]. In the case of IN drug delivery systems, several studies evaluating thiolated CS-based formulations have shown a significant enhancement of the drug’s nasal absorption, attributed to the protein tyrosinase and P-glycoprotein efflux pump inhibition in the mucosal membranes [38]. In this direction, a recent study regarding GAL IN administration using thiolated CS NPs has shown significant results (tested in amnesia-induced mice) [39].

A recent study evaluating a new nano-system, comprising GAL along with a novel hierarchical porous carbon (HPC) material, showed promising results regarding the transportation of the drug to the hippocampus within only few hours of a single IN administration [40]. These new findings provide a new way of utilizing carbon-based materials and the application in GAL IN formulations. In this vein, carbon nanodots (CDs), an emerging ultrasmall (less than 10nm) zero-dimensional carbon nanomaterial, have shown promising results in terms of IN delivery [41,42], providing a class of ideal properties for these types of applications, such as a robust spherical size, excellent water solubility, low cytotoxicity and advantageous biocompatibility, as well as photochemical stability [43]. Hence, CDs are attractive drug-carrier nanomaterials, possessing bioimaging properties for theragnostic applications, and could provide a new approach to GAL’s successful IN delivery. Based on these findings, it seems that the combination of thiolated CS and suitable carbon-based materials may be a successful solution to GAL’s efficient IN administration.

Therefore, the aim of the present study was to evaluate, for the first time, the preparation of GAL IN NPs using cysteine-modified CS (Cys-CS), along with suitable carbon-based materials (HPC and CDs), as suitable drug matrix carriers. A schematic representation of the entire NPs’ design is pictorially shown in Figure S1 (Supplementary Material).

## 2. Results and Discussion

### 2.1. Cys-CS Characterization

**Figure 1:**
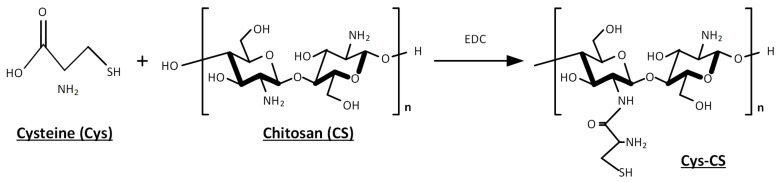
Reaction scheme for the preparation of a conjugated Chitosan (CS with L-cysteine (Cys).

Despite its various benefits, as stated in the introduction, CS is only soluble in acidic environments, a feature that limits some of its potential medical applications when administrated though routes with a physiological pH and/or moderately alkaline environmental (such as the nasal cavity). As a result, CS chemical modification is a potent tool for promoting novel biological activities and characteristics. In the present study, the modification of CS and the preparation of the Cys-CS conjugate was performed through amide bond formation between the free primary amino groups of CS and the carboxyl groups of Cys, as shown in the reaction scheme presented in Figure 1.

After the completion of the reaction and the removal of the remaining water used for washing, the obtained conjugate was received as a white-to-off-white, odorless powder.

#### 2.1.1. H-NMR

**Figure 2:**
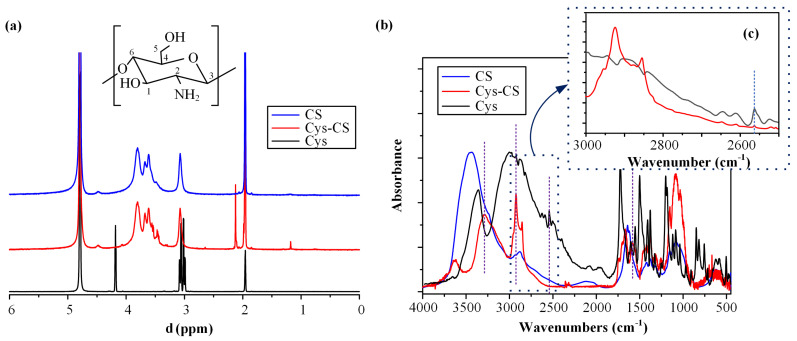
^1^H-NMR (**a**) and FTIR (**b**) spectra of the neat CS, the neat Cys and the conjugated Cys-CS; FTIR (**c**) spectra of the stretch vibration of the S–H bonds.

Figure 2a shows the ^1^H-NMR spectrum of the raw materials and the conjugated CS (Cys-CS).

Regarding the pure CS, the proton assignment based on the obtained ^1^H-NMR spectra showed several peaks at 1.91 ppm (due to the CH_3_ of the acetyl group), 2.88 ppm (due to the carbon 2), 3.5–3.8 ppm (due to carbons 3–6) and 4.6 ppm (due to carbon 1 of the polymer). Similarly, Cys showed a characteristic peak at 4.18 ppm, among others (at 4.79, 2.97–3.11, and 1.95 ppm). Looking at the ^1^H-NMR of Cys-CS, two new characteristic peaks were recorded at 2.12 and 1.79 ppm, respectively, which were not present in either neat component, indicating that the linkage (i.e., conjugation) between the CS and Cys occurred during the reaction. Furthermore, two small new peaks were recorded at 2.78 ppm and 3.12 ppm, respectively. The former was attributed to the methylene protons on -CH_2_SH, while the latter (appearing as a shoulder) was attributed to the proton –CH of the l-cysteine. Both these two new peaks indicate successful Cys-CS conjugation [44].

#### 2.1.2. FTIR Spectroscopy

The conjugation of the CS with Cys was further evaluated using FTIR. Figure 2b shows the FTIR spectrum of the raw materials and the modified CS (i.e., Cys-CS). Regarding the neat Cys, the obtained FTIR spectrum revealed several characteristic peaks in the region of 3000–2500 cm⁻^1^, attributed to the stretch vibration of the S–H bonds, while another characteristic peak at 1747 cm⁻^1^ was also recorded, corresponding to the carboxyl groups of the substance. Similarly, in the case of neat CS, a broad FTIR peak was recorded at 3400 cm⁻^1^, corresponding to the O-H and N-H stretching of the polymer, two peaks at 1641 and 1558 cm^−1^, attributed to the amide I and II respectively, a peak at 1324 cm⁻^1^, corresponding to the axial deformation of the C–N bond, and two peaks at 1074 and 1026 cm^−1^, attributed to the C–O bond of cyclic alcohols and the primary hydroxyl groups of the polysaccharide, respectively. Finally, the FTIR spectrum of the Cys-CS showed some additional signals (as compared to the neat CS), located at 1570 and 2927 cm⁻^1^, respectively, while the broad band CS corresponding to the O-H and N-H stretching of the polymer moved in lower wavenumbers (i.e., from at 3400 to 3723 cm⁻^1^). Additionally, a very weak IR peak at 2543 was recorded, and assigned to the S-H stretching vibration [45]. These new peaks, along with the aforementioned peak shift, confirm the conjugation between the two compounds. Similar results were previously reported regarding the FTIR spectrum of the Cys-CS and its successful conjugation [46].

#### 2.1.3. Degree of Deacetylation (DD)

The presence of primary amino groups in the backbone of CS (i.e., its DD) explains the flexibility of CS’s functionalization. These amino reactive groups provide the necessary sites for side group attachment (i.e., conjugation). Hence, evaluating the degree of deacetylation after a conjugation reaction provides a way to estimate the degree of amino substitution, and, therefore, the degree of conjugation.

**Figure 3:**
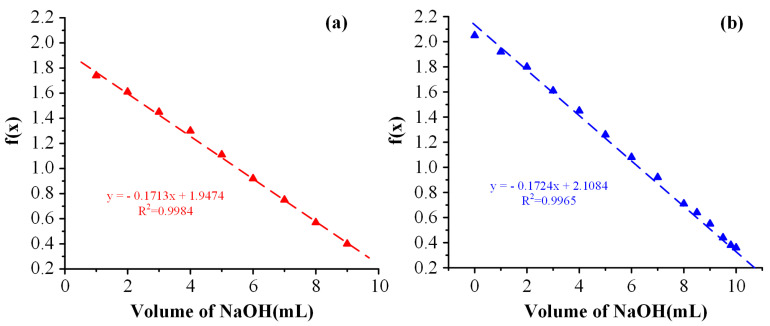
Linear titration curves of CS (**a**) and Cys-CS (**b**) used for the estimation of DD.

In the present study, the DD was estimated based on the potentiometric titration approach. The linear titration curve in the neat CS and the Cys-CS, along with the respective fitting equations and the coefficient of determination (R^2^), are shown in Figure 3.

The %DD, calculated from Equations (2) and (3), for the neat CS and Cys-CS, were 90.74% and 83.43%, respectively, indicating that a ~7.3% modification occurred during the Cys-CS conjugation procedure.

#### 2.1.4. Physical State Evaluation via pXRD

**Figure 4:**
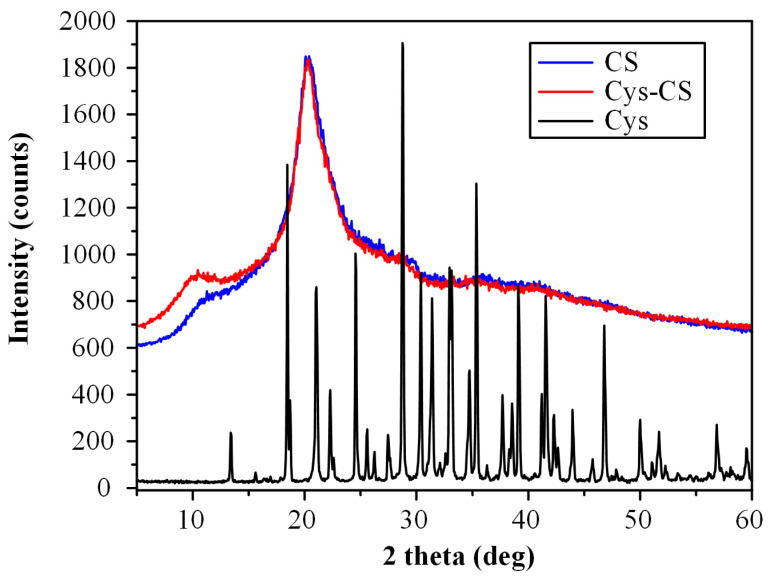
pXRD diffractograms of the neat CS, the neat Cys and the conjugated Cys-CS.

The physical state of the prepared conjugate was evaluated via pXRD. Figure 4 shows the pXRD diffractograms of the initial raw materials (i.e., CS and Cys-CS) and the conjugated Cys-CS.

Regarding the neat raw materials, the results showed that CS is a semi-crystalline polymer with two characteristic diffraction peaks, recorded at 2θ of 11.0 and 21.0 deg, while Cys is a highly crystalline compound with sharp pXRD peaks at 13.5, 18.4, 21.5, 24.6, 28.8, 30.4, 31.4, 33.0, 35.3, 39.0 and 41.6 deg. Similarly, the conjugated Cys-CS showed the two same characteristic diffraction peaks as the neat CS, indicating that the prepared modified CS was also semi-crystalline in nature.

#### 2.1.5. Solubility Result

**Figure 5:**
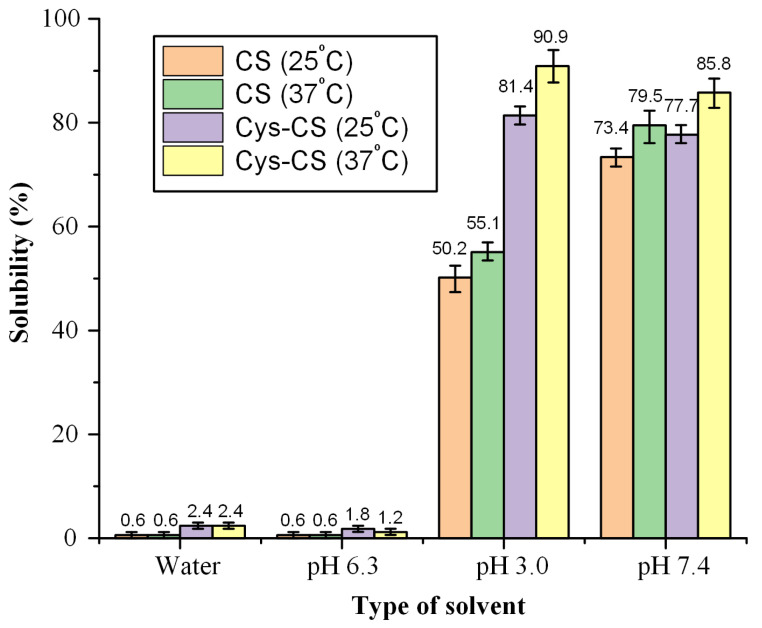
Solubility of CS and Cys-CS at 25 and 37 °C.

As stated earlier, CS-limited solubility has limited applications in biopharmaceutical applications, and especially during IN administration. Hence, to evaluate whether the proposed modification is actually able to improve the solubility profile of the neat polymer, the solubility of the neat CS and the Cys-CS conjugate in different solvents was evaluated. The results, presented in Figure 5, show that the solubility of the conjugated Cys-CS in water and in pH 6.3 (which is the close to nasal mucosal pH) increases by more than 100% as compared to the neat CS (i.e., from ~0.6% for the neat CS to > 1.2% for Cys-CS). Similarly, although not to the same extent, the solubility of the newly prepared conjugate significantly increases as compared to the neat CS in both pH 3.0 and in pH 7.4. Analogous observations were made by Hakimi et al. [47], who found that CS-Cys zeta potential was greater at acidic and physiological pH compared to CS, revealing an increase in the solubility of the CS-Cys derivative, while Liu et al. [48] synthesized a similar water-soluble thiolated chitosan derivatives. The increased solubility in the case of Cys-CS is due to the presence of the hydrophilic functional groups of Cys that interrupt the intermolecular H-bond and crystalline structures of CS, resulting in a significant enhancement of solubility. Hence, based on the obtained results, it is obvious that the proposed modification significantly improves CS’s solubility and, therefore, its utility as a matrix-carrier for drug formulations designed for IN applications. Pictures of the CS precipitation and the improved solubility of Cys-CS at alkaline pH are presented in the Supplementary Material (Figure S2).

### 2.2. Characterization of GAL-Loaded NPs

The newly synthesized cys-CS was used for the preparation of GAL-loaded NPs that were previously encapsulated in either CDs or HPC. Hence, several NP formulations were prepared with the neat GAL, the GAL-CDs and the GAL-HPC incorporated in the cys-CS. The same set of samples were also prepared using neat CS, instead of the cys-CS, in order to evaluate the role of cys conjugation in the performance and characteristics of the drug-loaded NPs.

#### 2.2.1. Drug Loading, EE and NP Yield

Table 1 summarizes the drug loading, EE and NP yield results for the prepared formulations.

Based on the obtained results, drug loading was higher in the case of GAL-loaded HPC nanostructures as compared to CDs, while no significant changes were observed for entrapment efficiency and nanoparticle yield. The concerning drug-loading results are probably due to the larger pores of HPC, as well as its larger surface area, which can retain greater quantities of the drug, thus leading to higher drug-loading values. Additionally, results showed slightly higher drug loadings when the conjugated Cys-CS was used as compared to the neat CS in all cases. One possible reason for this enhancement is that the Cys-CS conjugated polymer forms a more complexed structure (as compared to the neat CS), within which the drug is more effectively trapped and, hence, strongly restrained during the NPs’ evaporation and cleaning processes. Regarding EE and yield, results from the same table showed a similar encapsulation ability production efficiency in all prepared NPs, with EE values varying from 36.54% to 42.36%, and yield from 18.22% to 21.24%. It is important to note that the rather low values recorded for EE and yield can be attributed to the low batch size used during such small-scale trials (leading to high drug losses) or to the multi-step drug-loaded NP preparation method followed in the present study (i.e., the API was initially loaded into the HPC and CDs, and then this new binary system was loaded into the NPs), which may also lead to high drug losses.

#### 2.2.2. NPs’ Morphology via SEM

**Figure 6:**
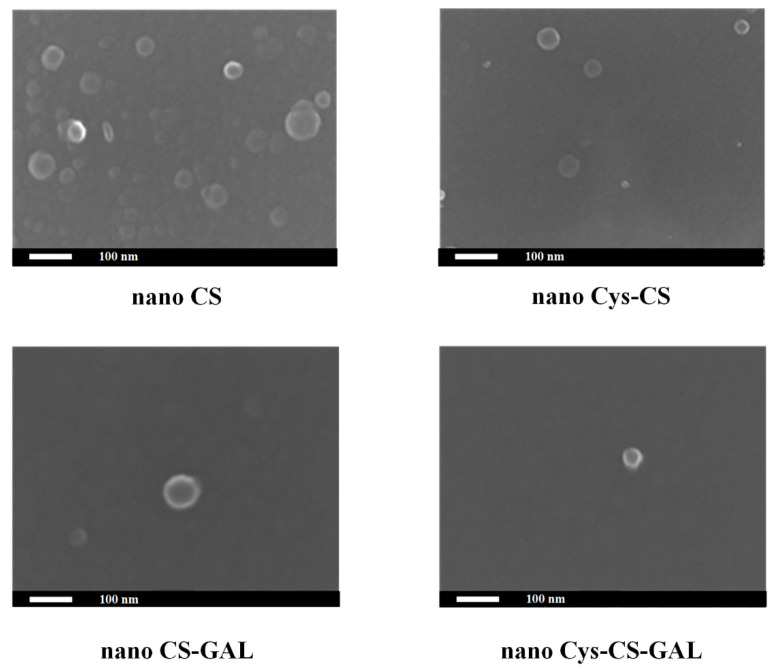
SEM images of neat and drug-loaded CS and Cys-CS NPs.

In a further step, the morphology of the prepared NPs was evaluated through SEM analysis. Figure 6 shows the SEM images of the CS-GAL and Cys-CS-GAL NPs, utilizing both the pure neat CS and the modified conjugate (Cys-CS).

Based on the obtained results, both NPs were spherical in shape, with a smoothed external surface. Furthermore, no agglomeration was observed in all formulations. Their size, based on the obtained SEM images, varied from approximately 40 up to approximately 80 nm. A more thorough analysis and comparison of their PSD differences is conducted below.

#### 2.2.3. PSD and ζ-Potential Results

The PSD and ζ-potential of the prepared NPs was evaluated via DLS. Table 2 summarizes the PSD values (along with the polydispersity index (PDI)), as well as the ζ-potential values of all prepared NP systems.

Based on the obtained results, the particle size of the neat NPs (i.e., those that did not contain the active pharmaceutical ingredient (API)) varied from ~320 to ~500 nm, with Cys-CS based NPs being significantly larger compared to the neat CS. Additionally, the encapsulation of GAL resulted in NPs having slightly larger particles as compared to the neat NPs. Regarding the NPs prepared with the addition of the new carbon-based materials (i.e., HPC and CD), the results showed a significant increase in the size of the resultant particles, reaching, in some cases, values above 1.0 μm. However, looking more closely into the obtained results, it seems that the use of CD, instead of HPC, leads to NPs with lower particle sizes, irrespective of the CS polymer used (i.e., CS or Cys-CS), while, in all cases, the size of the prepared CD-based NPs was below 850 nm. Hence, it can be said that the prepared CS-based GAL formulations only occur in the form of nano-sized particles when CD is used as the carbon-based material.

It is important to note that, based on the obtained DLS results, it seems that, after drug loading, the particle size of the prepared NPs varied from ~800 to ~1 mm, while the PDI values in all cases were also quite high (probably due to the formation of agglomerates) for intranasal administration. However, given the SEM images, the formed NPs were significantly lower in size (below 100 nm), while no agglomerates were seen (at least in the examined sample), which makes the use of these NPs suitable for intranasal administration. Various explanations for the differences in these SEM and DLS may include: (1) the fact that SEM is a 2D representation of a 3D particle and, hence, SEM assumes that these are discs to plot a circular equivalent; (2) in DLS, any conversions to a diameter involve the use of Stokes–Einstein equation with an assumption of shape; (3) DLS examines the entire particle (not just the electron-rich core), including protective surfactants and stabilizers; (4) DLS is an ensemble method, looking at the entire distribution (including agglomerates), and does not count particles; (5) DLS provides a distribution based on the scattering intensity of particles and, thus, the larger particles (agglomerates) are more important in such a distribution; (6) any mass/volume/intensity distribution given by DLS will always be larger for a polydisperse sample (such as those prepared in the present study). However, it should be pointed out that, in order to obtain a safe conclusion regarding the size/distribution of the prepared NPs (and their suitability for intranasal administration), an in-depth evaluation, with more sophisticated organology (for example with cryo-TEM), is needed. Nevertheless, for the purpose of the present study (which is a proof-of-concept study), the obtained results seem to be promising.

In a further step, besides the particle size, ζ-potential was also evaluated for all prepared NPs. In general, ζ-potential presents an indirect measurement of colloidal suspension’s stability, with values between ₋10 and +10 mV showing rapid agglomeration and, thus, long-term instability. In the present study, all tested formulations showed ζ-potential values above 40 mV, indicating a reduced tendency of agglomeration and, consequently, destabilization. Similar results of high positive ζ-potential values were also reported for CS-based NPs prepared with the same manufacturing process (i.e., via ionic gelation) [49].

#### 2.2.4. Evaluation of Drug’s Crystalline State after NP Production via pXRD

**Figure 7:**
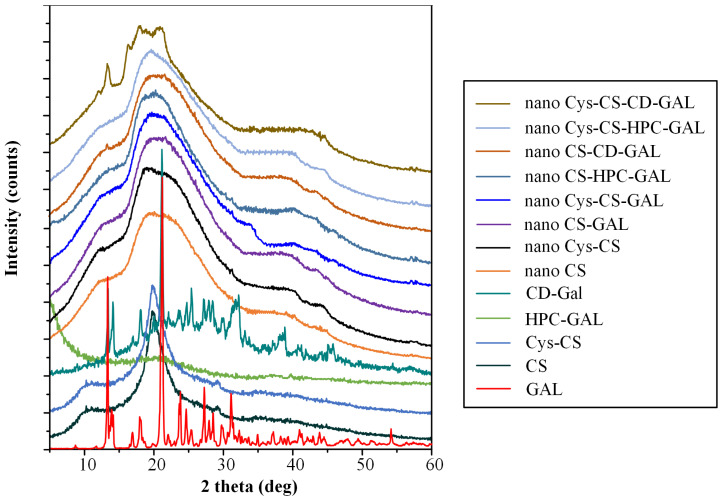
pXRD diffractograms of raw materials and the prepared NPs.

Changes in the crystalline state of the raw materials during the preparation of the NPs was evaluated via pXRD. Figure 7 shows the pXRD diffractograms of the raw materials and the prepared NP formulations using both CS and Cys-CS.

Regarding the physical state of GAL after it was loaded into the selected carbon materials (i.e., HPC and CD), the results showed significant differences. Specifically, when the drug was loaded into the HPC, no GAL diffractogram peaks were recorded, indicating that the API was amorphously dispersed into the highly porous network of the matrix-carrier. However, the pXRD diffractogram of the GAL-loaded CDs showed all characteristic API pXRD peaks, indicating that, during the preparation of GAL-CD, the API recrystallized. Hence, according to the obtained results, it seems that the selection of carbon-based materials significantly affects the physical state of the drug, with CD results being more promising, since the drug, in this case, is thermodynamically stable (i.e., crystalline). Regarding the prepared NPs, the obtained diffractograms showed that, in almost all cases, the drug was amorphously dispersed, since only the broad pXRD peak i CS or Cys-CS was recorded, along with a characteristic amorphous halo. The exemption to this was the NPs utilizing the CDs, where several characteristic GAL pXRD peaks were recorded, irrespective of the type of polymer (i.e., CS or Cys-CS), indicating that the drug, in these cases, was crystalline.

#### 2.2.5. Evaluation of Molecular Interactions after NP Production via FTIR Spectroscopy

**Figure 8:**
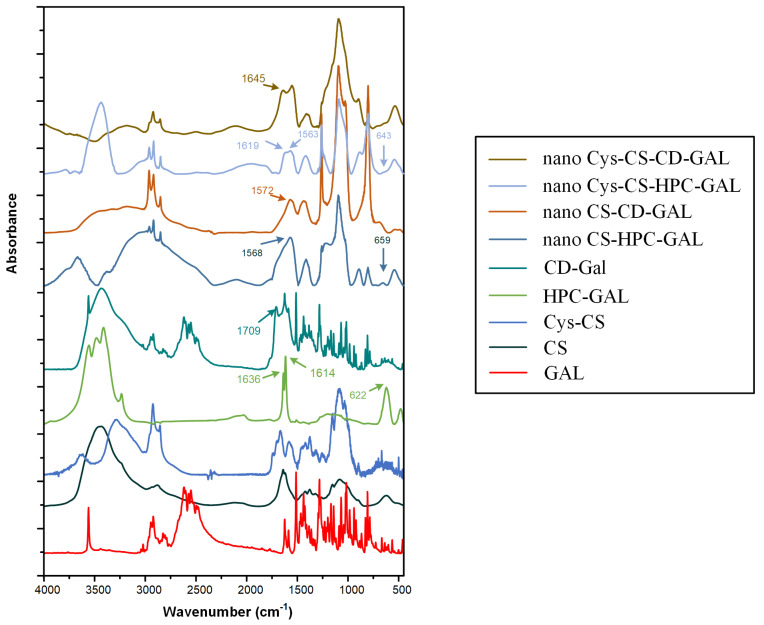
FTIR spectra of the raw materials and the prepared NPs.

The formation of molecular interactions between the drug and the tested NP matrix-carriers was evaluated with the aid of FTIR spectroscopy. Figure 8 shows the FTIR spectra of all components and the prepared NP formulations.

Regarding the pure GAL, the obtained FTIR spectrum revealed several characteristic vibrational peaks. The most distinctive were those located at 2830 cm⁻^1^, which were attributed to the CH stretching vibration, 3417 cm⁻^1^ corresponding to the NH stretching, and 3555 cm⁻^1^ corresponding to the OH vibrations. Looking at the spectra of the drug-loaded CS NPs, the most dominant peaks were those corresponding to the polymeric matrix-carrier (CS), such as the peaks at 2884, 1641 and 1074 cm⁻^1^, corresponding to the stretching vibrations of the CH groups, the amide I and the C–O bond of cyclic alcohols, and the primary hydroxyl groups of the polysaccharide, respectively. However, a closer look at the recorded spectra reveals characteristic peaks in GAL as well (i.e., the sharp peak at 3555 cm⁻^1^), while several peaks were shifted in either lower or higher wavenumbers compared to the neat API (for example, the peak at 1510 cm⁻^1^ was shifted at 1496 cm⁻^1^, while the peak at 1436 cm⁻^1^ shifted to 1440 cm⁻^1^). Furthermore, compared to the neat raw materials, a much broader peak at ~3400 cm⁻^1^ was recorded in the spectrum of the CS-based NPs (corresponding to the OH group vibrations), indicating that molecular interactions, in the form of H-bonds, are likely evolving between the two components. Similarly, as in the case of CS-based NPs, the same FTIR peak shifts were also recorded in the case of Cys-CS NPs, indicating the formation of a similar H-bond between the API and the conjugated matrix-carrier.

Regarding the GAL-loaded NPs using HPC as a porous carbon matrix-carrier, in addition to the polymer (CS or Cys-CS) and the API characteristic FTIR vibrational peaks, several peaks corresponding to the HPC were also recorded, such as the two peaks at 1616 and 1639 cm⁻^1^, corresponding to the stretching of the C=C bonds, the three peaks at 3416, 3477 and 3553 cm⁻^1^, due to >C–H bonds’ vibration, and the peak at 3235 cm⁻^1^, corresponding to the stretching vibration of the OH located on the surface of the HPC. However, a closer look at the obtained spectra reveals that several peaks corresponding to the API were shifted to lower wavenumbers, such as the peak at 1636 cm⁻^1^ (which shifted to 1616 cm⁻^1^) and the peak at 1614 cm⁻^1^ (which shifted at 1563 and 1568 cm⁻^1^, in the case of NPs having CS and Cys-CS as matrix polymers, respectively), indicating that molecular interactions (probably H-bonds) were also present when the HPC was added to the system, irrespective of the type of CS used (wither neat or conjugated with Cys). Similar results (i.e., the FTIR peak shifting presented in Figure 8) were also recorded in the case of CDs-based NPs, indicating that significant molecular interactions were also present in these systems.

#### 2.2.6. In Vitro Dissolution Release Results

**Figure 9:**
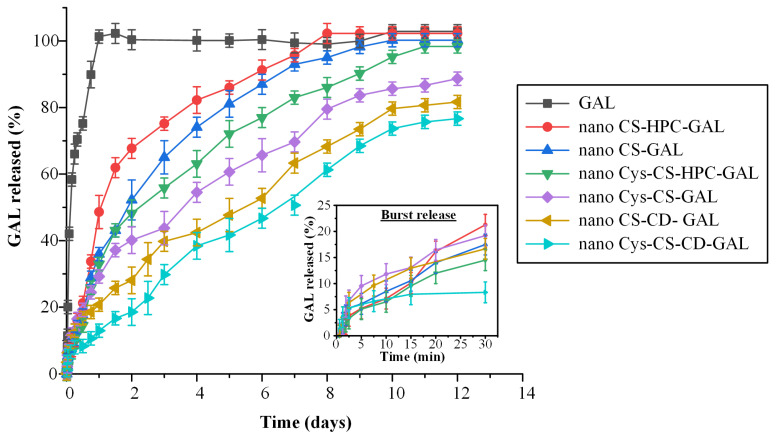
In vitro dissolution profiles of the pure GAL and the prepared NPs.

Figure 9 shows the in vitro dissolution profile of all prepared NPs, as well as the same profile for the pure API. 

Regarding the neat GAL, the results showed an immediate release profile, with 100% of the drug being released within the first hour. This was expected, as sink conditions were maintained during dissolution testing. Regarding the prepared NPs, analysis of the dissolution results reveals a biphasic release profile in all cases. Specifically, an initial burst release phase (phase I) was recorded in all formulations until approximately 15–30 min (phase I), which can likely be attributed to surface-absorbed GAL. Then, after the initial burst, a steady, sustained drug release phase (i.e., phase II) was observed, which was completed in approximately 12 days, associated with the swelling (i.e., gel formation) and biodegradation (i.e., erosion) mechanisms of the polymeric carriers that were used (i.e., CS and Cys-CS).

Looking more closely at the 1st phase of the dissolution process (embedded graph in Figure 9), it seems that all prepared NPs show similar initial burst release characteristics, although it is obvious that, in the NPs with CD and Cys-CS, the API released within the first 30 min was significantly lower (~7.5%) compared to the rest of the formulations (~14–22%). Regarding the second (sustained) release phase, the results clearly show that the NPs prepared with the conjugated Cys-CS presented a slower release rate compared to those manufactured with the neat CS. This indicates that, due to the presence of the thiol groups in the backbone of the conjugated polymer, stronger intermolecular interactions were formed between the polymer and the API, thus postponing its solubilization during dissolution. Additionally, when HPC was used in the preparation of drug-loaded NPs, fast dissolution rates were obtained in all cases, while the use of CD resulted in remarkably slower drug release rates. An explanation of this prolonged delivery when CD was used may be given by the diffractograms presented in Figure 7, where the API recrystallized in the presence of CD, while it remained amorphous in the rest of the cases. Hence, the amorphicity of the API presented in the aforementioned systems probably led to its faster wetting and, consequently, its faster solubilization and dissolution from the prepared NPs.

In addition to the above, it is important to note that the release of the API from the prepared NPs is also controlled by the specific characteristics of the polymer during its wetting and solubilization process, i.e., its swelling and erosion/degradation abilities. Hence, it can be assumed that at least two different mechanisms prevail during GAL’s solubilization and, thus, in order to evaluate their effect on the API’s dissolution profile the obtained dissolution data were fitted in various kinetic models (Equations (7)–(11)). Table 3 summarizes the goodness of fit (correlation coefficient, R^2^) and the k-constants for each model.

The results showed that the Korsmeyer–Peppas model had higher R^2^ values in all cases, indicating that the aforementioned model is more suited to describing the release mechanism of the drug from the prepared NPs. Generally, by employing the exponent *n*, the Korsmeyer–Peppas model characterizes the several mechanisms determining drug dissolution features in such formulations, with *n* values < 0.5 indicating that the API is released via a quasi-Fickian diffusion mechanism, while values between 0.5 and 1 suggest an anomalous, non-Fickian, drug diffusion [50]. Looking at the obtained results, the *n* fitting exponent parameter was, in most of the cases, below 0.5 indicating that the release of the API was diffusion-controlled. Interestingly, in the case of CD-based NPs, the Korsmeyer–Peppas fitting exponent parament, *n*, increases above 0.5, suggesting that, in this case, the mechanism of GAL release changes from quasi-Fickian to anomalous, non-Fickian, drug diffusion. What is more interesting to note is that, in the case where both CDs and the conjugated Cys-CS were used simultaneously, the obtained GAL dissolution profile reaches an almost perfect zero-order release profile (this is also seen by the high R^2^ obtained for the zero-order release model fitting). This is an indication that a balance between diffusion-controlled and matrix erosion leads to a zero-order release profile, which is essential to achieving stable in vivo pharmacokinetic behavior.

## 3. Materials and Methods

### 3.1. Materials

GAL (C_17_H_21_NO_3_; molecular mass: 287.35 g/mol) was kindly donated by Pharmathen S.A. (Athens, Greece). Low molecular CS (5–19 kDa) was purchased from Aldrich Chemical Co. (Stainheim, Germany). L-cysteine was purchased from Serva (Heidelberg, Germany). N-(3-Dimethylaminopropyl)-N′-ethylcarbodiimide hydrochloride (EDC), N,N-dimethylformamide (DMF), ethanol absolute, colloidal silica, sucrose, and sodium tripolyphosphate (TPP), were purchased from Sigma-Aldrich (St. Louis, MO, USA). All other chemicals were of analytical grade, while all solvents used in HPLC analysis were of HPLC grade.

### 3.2. Synthesis of Materials and NPs

#### 3.2.1. Synthesis of Cys-CS

For the preparation of Cys-CS, a modification of a previously published protocol was followed [51]. In brief, 2g of CS powder was dissolved in 250 mL of 1% (*v*/*v*) acetic acid aqueous solution overnight for complete solubilization. Then, 0.3967 g of EDC was added, along with 2 g of L-cysteine dissolved in 40 mL of water. The solution was left at 50 °C under magnetic stirring (750 rpm) for three days. Next, the pH was adjusted to 7 and the resultant suspension was centrifuged at 4000 rpm for 15 min. The precipitate was collected, washed with water twice and then freeze-dried for 12 h at −56 °C to remove any remaining water in a Scanvac freeze-drier (Scanvac Coolsafe, Labogen Scandinavia, Lillerød, Denmark). The resulting Cys-CS, in the form of powder, was hermetically sealed and placed in a desiccator until further use.

#### 3.2.2. Synthesis of HPC

The synthesis of HPC was performed based on a previously described procedure [40,52]. In brief, colloidal silica was initially suspended in water (40% *w*/*w*) and then mixed with sucrose at a silica-to-sucrose ratio of 2/1 *w*/*w*. The resultant suspension was casted into liquid N_2_, and then freeze-dried for 2 days using a Scanvac freeze-drier (Scanvac Coolsafe, Labogen Scandinavia, Lillerød, Denmark). Carbonization of the obtained solid powder was performed under a continuous flow of N_2_ gas (1050 °C for 3 h), while etching of the silica template was achieved via a solution of 3M sodium hydroxide. Finally, after washing the resultant sample with water for several times, micropores were generated by activation under CO_2_ gas flow at 950 °C.

#### 3.2.3. Synthesis of CDs

In the present work, CDs were synthesized based on a previous study [53]. In brief, CDs were prepared by dissolving citric acid and urea (1/2 *w*/*w* ratio) in DMF. The reaction was conducted in solvothermal condition at 160 °C for 5 h. The product was precipitated drop by drop in ethanol and collected by centrifuging at 12,000 rpm for 30 min, and then it was dispersed in ultrapure water by sonication.

#### 3.2.4. HPC Drug Loading Process

In order to load the API into the HPC, 50 mg of GAL were dissolved in 100 mL of methanol. Then, an accurately weighted quantity of the prepared HPCs was transferred to the solution and left under magnetic stirring for 24 h. The resultant suspension was centrifuged at 12,500 rpm for 20 min and the precipitate was collected and dried in an oven at 40 °C for 4h, in order to remove any excess methanol. The collected drug-loaded HPCs were hermetically sealed and placed in a desiccator until further use.

#### 3.2.5. CDs Drug Loading Process

A similar procedure to 3.2.4 was followed to load the API to CDs.

#### 3.2.6. Preparation of NPs

The preparation of GAL-loaded NPs was conducted based on the ionic gelation method presented elsewhere [54]. In brief, 100 mg of CS or Cys-CS were dissolved in 25 mL of 2% *v*/*v* acetic acid solution. Then, 5 mg of GAL, GAL-loaded HPCs or GAL-loaded CDs were dispersed with the aid of a probe sonicator for 1 min at cycle 1 and amplitude 100% (Germany, Hielscher Ultrasound Technology, Model UP100H). A total of 12.5 mL of TPP (dissolved in water at a 2 mg/mL concentration) was added and the resultant suspension was left under mild magnetic stirring for 24 h. The obtained NPs were isolated by centrifugation at 12,500 rpm for 20 min and washed with water. The resultant NPs were finally freeze-dried and stored at 4–8 °C until further use.

### 3.3. Characterization

#### 3.3.1. Characterization of HPC

The newly synthesized HPCs were fully characterized in a recently published study of ours [40]. In brief, the prepared carbon materials possessed a high surface area (2200 m^2^/g) and pore volume (4.014 cm^3^/g), while, during its preparation, the sublimated ice crystals were fully replaced from macropores.

#### 3.3.2. Characterization of CDs

Similarly, the characterization of the synthesized CD has been provided in a recently published study [55]. In brief, the CDs were synthesized using the microwave-assisted pyrolysis procedure using citric acid and urea [56]. The results revealed the successful formation of spherical size carbon nanostructures with a diameter of approximately 4.5 nm and carbon functionalities on the surfaces, such as C-O, C-N and C=O bonds.

#### 3.3.3. Characterization of Cys-CS

Nuclear Magnetic Resonance Spectroscopy (^1^H-NMR): ^1^H-NMR spectra of Cys-CS, L-cysteine, and CS (all in 5% w/v) were recorded in deuterated chloroform (CDCl_3_), on an Agilent 500 spectrometer (Agilent Technologies, Santa Clara, CA, USA) at room temperature. The number of scans was 16 and the sweep width was 6 kHz.

Fourier-Transformed Infrared (FTIR) Spectroscopy: FTIR spectra were collected on an FTIR spectrometer (model FTIR-2000, Perkin Elmer, Dresden, Germany) by using KBr discs (thickness of 500 μm). The spectra were collected in the range from 4000 to 400 cm⁻^1^ at a resolution of 2 cm⁻^1^ (total of 20 co-added scans). The presented spectra were baseline corrected and converted into absorbance mode.

Powder X-Ray Diffractometry (pXRD): pXRD analysis of samples was performed in Rigaku XRD-diffractometer (Miniflex 600, Chalgrove, Oxford, UK) with a CuKα radiation for crystalline phase identification (λ = 0.15405 nm for CuKα). All samples were scanned from 5° to 40° with a step scan of 0.05 deg, and 1 deg/min scan speed.

Solubility: Solubility of CS and Cys-CS was evaluated at 25 and 37 °C at several pH (i.e., pH = 3, pH = 6.3 and pH = 7.4) using the shaking flask method, based on a previously published methodology [57]. Double-distilled water was used to prepare the solution mediums. The pH of the solution medium was adjusted by preparing a 2% acetic acid solution (*v*/*v*). Then, aqueous NaOH solution (0.1 M) was added dropwise to the solution until the desired pH value was reached. For the solubility measurements, 0.05 g of the polymer (pure of modified) was placed in suitable flasks containing 3 mL of the aforementioned solution mediums and left under stirring for 48 h. The flasks were hermetically sealed in order to prevent any solvent evaporation. Then, the resultant suspension was centrifuged at 4000 rpm for 30 min and the supernatant was freeze-dried to remove any excess remaining water. The solubility of the polymer in each solution medium was calculated based on the quantity of the polymer collected after the freeze-drying process.

Deacetylation Degree (DD): The DD of the pure CS and the modified Cys-CS was evaluated using the potentiometric titration approach [58]. In brief, accurately weighted samples (∼0.25 g) of CS or Cys-CS were dissolved in an excess aqueous acidic solution (0.1 N HCl solution). This solution was then titrated with 0.1 M NaOH, measuring pH with a pH-meter. Titration was performed until the chitosan solution reached a pH of approximately 6.5 (range of chitosan non-protonation). All measurements were made in triplicate. An *f(x)* value, corresponding to the utilized NaOH volume, was calculated by the following equation:

(1) $$f\left(x\right)=\left(\frac{V_{0}+V}{N_{B}}\right)\times \left(\left[H^{+}\right]-\left[H^{-}\right]\right)$$

where *V*_0_ is the volume of the CS (or Cys-CS) solution, *V* is the volume of NaOH utilized in the titration, N_B_ is the concentration of NaOH, [H^+^] is the concentration of H^+^ and [OH−] is the concentration of OH^−^. The linear titration curve was obtained by plotting a graph of *f(x)* versus NaOH volume. The NaOH volume at the end of titration, *V_e_*, was found by extrapolating the linear titration curve as a function of the added NaOH volume. The DD was calculated from the following equations:

(2) $$DD\left(\%\right)=\frac{\varphi }{\left[\left(W-161\varphi \right)/204+\varphi \right]}\times 100$$

(3) $$\varphi =\frac{\left(N_{a}V_{A}-N_{B}V_{e}\right)}{1000}$$

where *N_A_* is the HCl concentration, *V_A_* is the HCl volume *N_B_* is the NaOH concentration, *V_e_* is the volume of NaOH at the end of the titration, and *W* is the sample mass

#### 3.3.4. Characterization of NPs

Drug loading, Encapsulation Efficiency (EE) and NP yield: GAL drug loading and EE was performed by assaying, using the HPLC method described below. For the preparation of the assay sample, 1.0 mg of NPs was dissolved in dichloromethane:methanol (1:1 *v*/*v*). Drug loading, yield, and entrapment efficiency were calculated using the following equations:

(4) $$\mathrm{Drug}\;\mathrm{loading}\;\left(\%\right)=\frac{\mathrm{weight}\;\mathrm{of}\;\mathrm{GAL}\;\mathrm{in}\;\mathrm{NPs}}{\mathrm{weight}\;\mathrm{of}\;\mathrm{NPs}}\times 100$$

(5) $$\;\mathrm{Yield}\;\left(\%\right)=\frac{\mathrm{weight}\;\mathrm{of}\;\mathrm{NPs}}{\mathrm{initial}\;\mathrm{weight}\;\mathrm{of}\;\mathrm{raw}\;\mathrm{materials}}\times 100$$

(6) $$\mathrm{EE}\;\left(\%\right)=\frac{\mathrm{weight}\;\mathrm{of}\;\mathrm{GAL}\;\mathrm{in}\;\mathrm{NPs}}{\mathrm{initial}\;\mathrm{weight}\;\mathrm{of}\;\mathrm{GAL}}\times 100$$

Dynamic Light Scattering (DLS): Particle Size Distribution (PSD) and ζ-potential were determined by dynamic light scattering (DLS) using a Zetasizer Nano-S system (Malvern Instruments, UK). For the preparation of samples, a 100-fold dilution with a low-ionic-strength (2 mM) phosphate buffer was performed at pH 7. All measurements were conducted at 25 °C and in triplicate.

Scanning Electron Microscopy (SEM): SEM images were acquired with an electron microscope JEOL 2011 (Akishima, Tokyo, Japan). For the measurements, a drop of each NP suspension was placed in the holder and left to evaporate. Samples were covered with carbon to provide a good conductivity of the electron beam. Operating conditions were set at an accelerating voltage of 20 kV, probe current of 45 nA and counting time of 60 s.

FTIR Spectroscopy and pXRD: FTIR and pXRD analyses of the prepared NPs were performed following the same experimental procedure as described in Section 3.3.2.

In vitro Drug Dissolution Studies: For the in vitro dissolution release studies, a DISTEK Dissolution Apparatus I (dissolution system 2100C, Distek, North Brunswick, NJ, USA) was used, equipped with an autosampler (Evolution 4300, Distek, North Brunswick, NJ, USA). The dissolution of the samples was performed using appropriate dialysis tubing cellulose membranes, placed inside the baskets. All tests were executed at 37 ± 1 °C with 50 rpm. The dissolution medium was 500 mL of simulated body fluid (SBF) at pH = 7.4. SBF was prepared by adding 8g NaCl, 0.2g KCL, 1.44g Na_2_HPO_4_ and 0.245g KH_2_PO_4_ in 1 L of distilled water. Two milliliters of aqueous solution were withdrawn from the release media at predefined time intervals (15, 30, 45 min and 1, 2, 4, 6, 8, 12, 18, 24, 36, 48, 72, 96, 120, 144, 168, 192, 216, 240, 264 and 288 h) and quantified via the HPLC method described below.

To evaluate the drug-release mechanism, in vitro dissolution results were fitted to the following release kinetics models [50]:

Zero-order model: D_t_ = D_0_ + k_0_t (7)

First-order model: logD_t_ = logD_0_ + k_1_t/2.303 (8)

Higuchi square root model: D_t_ = D_0_ + k_H_t^1/2^ (9)

Hixon-Crowell model: D_t_^1/3^ = D_0_^1/3^ - k_HC_t (10)

Korsmeyer–Peppas model: D_t_/D_∞_ = D_0_ + k_P_t^n^ (11)

where Dt is the amount of drug released at time t, D_0_ is the initial amount of drug released, Dt/D_∞_ is fraction of drug released at time t, k_0_ is the zero-order release constant, k_1_ is the first-order release constant, k_H_ is the Higuchi release constant, k_HC_ is the Hixson–Crowell release rate constant, k_p_ is the Peppas release constant, and n is the release exponent respectively.

HPLC Analysis: GAL was assayed via HPLC using a Shimadzu Prominence HPLC system (Shimadzu, Tokyo, Japan), consisting of a degasser (Model DGU-20A5, Tokyo, Japan), a pump (Model LC-20AD, Tokyo, Japan), an autosampler (Model SIL-20AC, Tokyo, Japan), a UV–Vis detector (Model SPD-20A, Tokyo, Japan), and a column oven (Model CTO-20AC, Tokyo, Japan). The analysis was performed with a C18 column (CNW Technologies Athena, 120 Å, 5 m, 250 mm 4.6 mm, Tokyo, Japan) at 25 °C. The mobile phase consisted of a 10 mM aqueous solution of KH_2_PO_4_ (with a pH of 3.5) and methanol in a ratio of 80/20 *v*/*v*, respectively. The flow rate was set at 1.0 mL/min, the injection volume was 10 L, and GAL was detected at 235 nm.

## 4. Conclusions

In the present study of Cys-CS based NPs, new carbon-based materials, such as HPC and CD, were prepared for the IN administration of GAL. the results showed that the conjugation of CS with Cys, as well as the incorporation of HPC and CD in the prepared drug-loaded NPs, significantly affects both their physicochemical characteristics and their in vitro dissolution behavior. Specifically, the use of the Cys-CS conjugate led to a significant solubility enhancement in neutral and alkaline pH values, improving CS’s utility as a matrix carrier for IN drug formulations, while the use of CD led to the formation of a thermodynamically stable drug dispersion (since the API recrystallized in the presence of CD), while a zero-order release profile was achieved, which is essential to attain a stable in vivo pharmacokinetic profile. However, despite their promising results, more studies are needed (such as toxicity, ex vivo and in vivo trials) in order to establish the applicability of the proposed systems in GAL’s IN administration.

## Figures and Tables

**Table 1:** Summary of drug loading, EE and NP yield results.

Sample	Drug Loading (%)	EE (%)	NPs’ Yield (%)
Nano-CS-GAL	14.49 ± 1.06	41.83 ± 1.42	20.59 ± 0.61
Nano-CS-HPC-GAL	11.03 ± 0.95	38.95 ± 3.49	19.35 ± 1.05
Nano-CS-CD-GAL	10.91 ± 0.86	36.54 ± 2.94	21.24 ± 0.35
Nano-Cys-CS-GAL	19.37 ± 1.54	42.36 ± 2.68	18.22 ± 0.64
Nano-Cys-CS-HPC-GAL	17.32 ± 0.86	40.69 ± 2.49	20.41 ± 0.38
Nano-Cys-CS-CD-GAL	16.83 ± 1.48	39.28 ± 1.95	19.06 ± 1.52

**Table 2:** Summary of NPs’ PSD, PDI and ζ-potential results.

Sample	Particle Size (nm)	PDI	ζ-Potential (mV)
Nano-CS	328.58 ± 2.29	0.92	54.6 ± 2.2
Nano-Cys-CS	508.91 ± 3.10	0.81	58.3 ± 1.9
Nano-CS-GAL	386.86 ± 1.37	0.96	41.9 ± 0.3
Nano-Cys-CS-GAL	527.24 ± 2.68	0.88	44.0 ± 1.1
Nano-CS-HPC-GAL	983.02 ± 4.61	0.94	51.6 ± 2.9
Nano-Cys-CS-HPC-GAL	1030.95 ± 5.98	0.91	*
Nano-CS-CD-GAL	792.31 ± 4.08	0.87	43.9 ± 0.9
Nano-Cys-CS-CD-GAL	828.84 ± 5.81	0.91	44.5 ± 1.3

* ζ-potential was not measured as the particle’s size was above 1μm.

**Table 3:** Dissolution data model fitting results for the employed drug release kinetic models.

Release Model	NPs’ Formulations											
	CS-GAL		Cys-CS-GAL		CS-HPC-GAL		Cys-CS-HPC-GAL		CS-CD-GAL		Cys-CS-CD-GAL	
	R^2^	k	R^2^	k	R^2^	k	R^2^	k	R^2^	k	R^2^	k
Zero order	0.87	9.03 d⁻^1^	0.90	8.63 d⁻^1^	0.80	9.09 d⁻^1^	0.93	7.38 d⁻^1^	0.97	6.67 d⁻^1^	0.99	6.56 d⁻^1^
First order	0.99	0.37 d⁻^1^	0.99	0.29 d⁻^1^	0.99	0.53 d⁻^1^	0.96	0.21 d⁻^1^	0.96	0.15 d⁻^1^	0.98	0.12 d⁻^1^
Higuchi	0.97	32.83 d⁻^1/2^	0.98	30.33 d⁻^1/2^	0.94	35.03 d⁻^1/2^	0.98	26.93 d⁻^1/2^	0.97	23.40 d⁻^1/2^	0.95	20.60 d⁻^1/2^
Hixson-Crowell	0.97	0.10 d⁻^1^	0.97	0.07 d⁻^1^	0.98	0.12 d⁻^1^	0.93	0.05 d⁻^1^	0.95	0.04 d⁻^1^	0.98	0.03 d⁻^1^
Korsmeyer-Peppas	0.99	34.59 d⁻^n^ (n = 0.47)	0.99	30.21 d⁻^n^ (n = 0.50)	0.95	40.60 d⁻^n^ (n = 0.42)	0.99	28.15 d⁻^n^ (n = 0.47)	0.99	20.87 d⁻^n^ (n = 0.56)	0.99	13.14 d⁻^n^ (n = 0.73)

## Data Availability

Data is contained within the article.

## Supplementary Materials

The following supporting information can be downloaded at: https://www.mdpi.com/article/10.3390/polym14194004/s1, Figure S1: Schematic representation of the entire NPs’ design; Figure S2: Pictures of the CS precipitation and the improved solubility of Cys-CS at alkaline pH (pH = 7.4); Table S1: Standards’ concentration and calculated area for the HPLC method for the determination of Galantamine; Figure S3: Calibration curve for the HPLC method used to determine galantamine’s content.
