# Supplementary material for: L-Cysteine Modified Chitosan Nanoparticles and Carbon-Based Nanostructures for the Intranasal Delivery of Galantamine
Source: Polymers (Basel). 2022 Sep 24;14(19):4004. doi: 10.3390/polym14194004 (PMC9571213; doi:10.3390/polym14194004)
Supplement: Supplementary file 1 [file polymers-14-04004-s001.zip › polymers-1922588-supplementary.pdf]

Supplementary Material

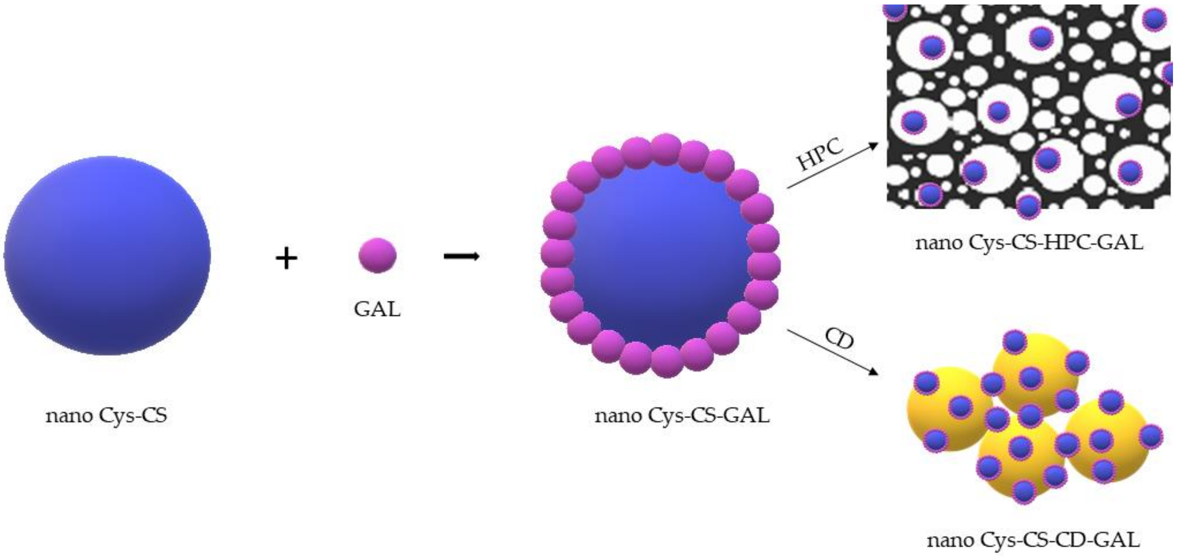

Figure S1: Schematic representation of the entire NPs' design

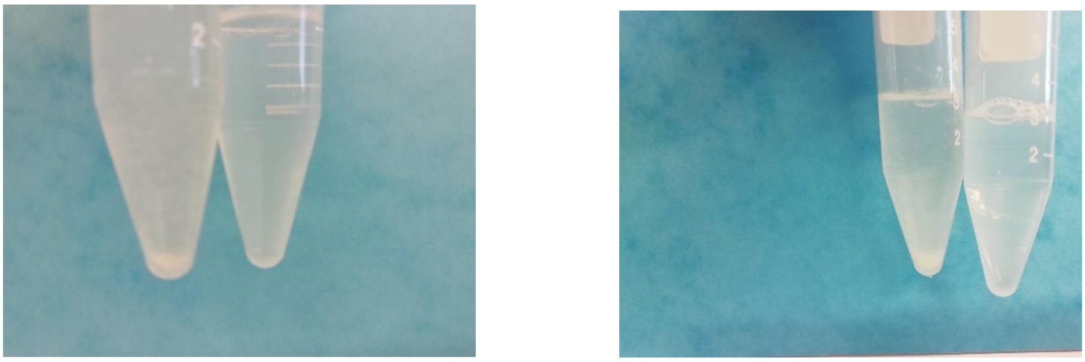

CS and Cys-CS at 25°C CS and Cys-CS at 37°C

Figure S2: Pictures of the CS precipitation and the improved solubility of Cys-CS at alkaline pH (pH = 7.4).

Table S1: Standards' concentration and calculated area for the HPLC method for the determination of Galantamine.

SAMPLE	CONCENTRATION (PPM)	AREA (RT 4.58MIN)
**STD_0.01**	0.01	308
**STD_0.05**	0.05	1333
**STD_0.1**	0.1	2962
**STD_0.5**	0.5	14686
**STD_1**	1	25227
**STD_2.5**	2.5	55220
**STD_5**	5	123590
**STD_10**	10	259840
**STD_20**	20	471632
**STD_50**	50	1170996

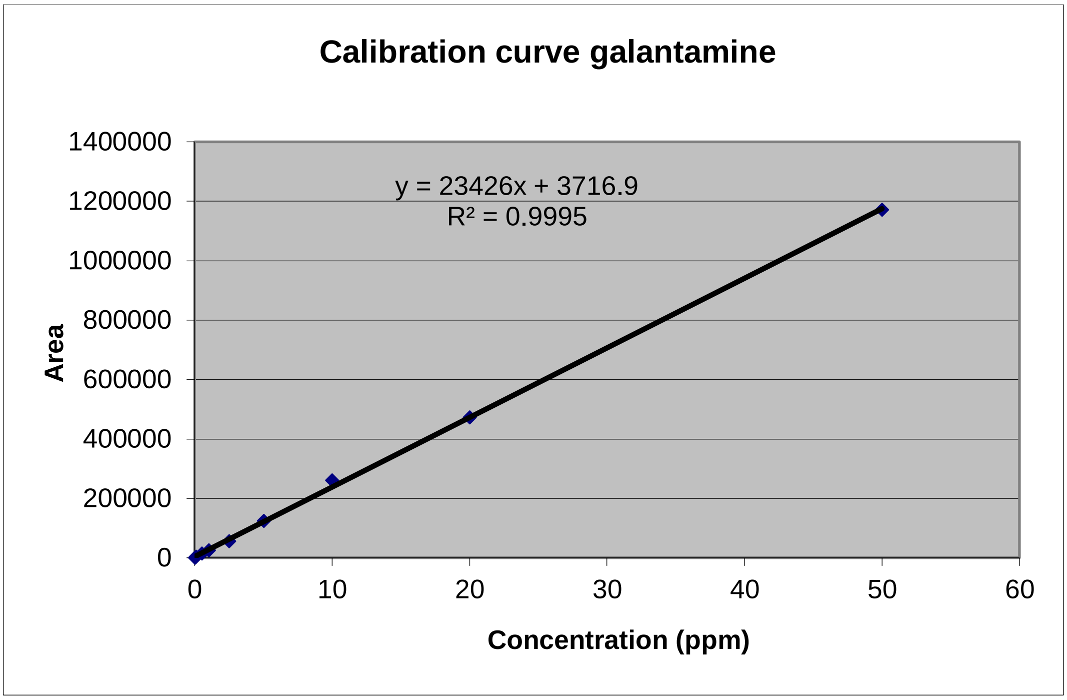

Figure S3. Calibration curve for the HPLC method used to determine galantamine's content.
